# A study of the value of requesting information from drug manufacturers for systematic reviews; 9 years of experience from the drug effectiveness review project

**DOI:** 10.1186/s13643-018-0834-2

**Published:** 2018-10-22

**Authors:** Marian S. McDonagh, Sujata Thakurta, Kim Peterson

**Affiliations:** 1 The Pacific Northwest Evidence-based Practice Center, Department of Medical Informatics and Clinical Epidemiology, Oregon Health and Science University, 3181 SW Sam Jackson Park Road, Portland, OR 97239 USA; 2 Department of Veterans Affairs, Health Services Research and Development, VA Portland Health Care System, 3710 SW U.S. Veterans Hospital Road, Portland, OR 97239 USA; 3 Department of Veterans Affairs, Evidence-based Synthesis Program (ESP) Coordinating Center, VA Portland Health Care System, 3710 SW U.S. Veterans Hospital Road, Portland, OR 97239 USA

**Keywords:** Systematic reviews, Publication bias, Reporting bias, Pharmaceutical manufacturers, Gray literature

## Abstract

**Background:**

Systematic reviews (SRs) depend on comprehensive searches for evidence to provide balanced, accurate results. Requesting published and unpublished studies from pharmaceutical manufacturers has been proposed as a method to engage industry stakeholders and potentially reduce reporting bias. The Drug Effectiveness Review Project (DERP) has been requesting such evidence since 2003; the purpose of this study was to retrospectively evaluate the type and impact of the evidence received.

**Methods:**

Data from “dossiers” submitted by pharmaceutical manufacturers for a set of 40 SRs conducted for DERP from July 2006 to June 2015 were retrospectively evaluated. Characteristics of data submitted in dossiers, including numbers, types, and characteristics of studies submitted and then included in DERP SRs, were abstracted. Time trends, study quality, publication status, and whether the submission represented a unique study or supplemental data to a published study were assessed. The impact of this evidence on SR conclusions was assessed using dual review. Differences were resolved through a consensus.

**Results:**

Over 9 years, 160 dossiers were received, relating to 40 DERP SRs. Out of 7360 studies/datasets submitted, 2.2% (160) were included in a SR. The ratio of submitted-to-included increased over time. Most were unique studies (23% were supplemental data sets), and almost 42% of the studies were unpublished. The majority of the studies were rated fair quality, with 7.3% rated good and 14% rated poor quality by the original SR authors. Considering all literature search sources, 7.2% of all studies included in the 40 SRs came from a dossier, and 16% of dossier studies were included in a meta-analysis. The dossier studies resulted in changes to conclusions in 42% of the SRs. Out of 46 unpublished unique studies included in a SR, 25 (54%) influenced the conclusions in favor of the manufacturers drug, 8% favored a competitor drug, and 40% favored neither. In 92% of cases favoring the manufacturer’s drug, the dossier study was the only evidence for that drug in a specific population or outcome.

**Conclusions:**

In SRs conducted for DERP, few studies submitted by pharmaceutical manufacturers were ultimately included in a SR. The included data helped to reduce reporting and publication bias by filling important gaps and in some cases led to altered conclusions.

**Electronic supplementary material:**

The online version of this article (10.1186/s13643-018-0834-2) contains supplementary material, which is available to authorized users.

## Background

A basic tenet of a systematic review (SR), in fact a defining characteristic, is the comprehensive nature of the search for evidence. This includes searching a variety of general biomedical and specialty bibliographic database sources, such as MEDLINE, to identify the most easily accessible published studies [1, 2]. Existing guidance on how to design the literature search for SRs includes specific advice on designing bibliographic database searches to identify certain types of studies, for certain databases, and so on, as well as more broad guidance from organizations such as the Agency for Healthcare Research and Quality’s (AHRQ) Evidence-based Practice Center (EPC) and the Cochrane Collaboration [1, 2].

To minimize publication bias, or other reporting biases, SRs must also search numerous other sources to seek out gray literature (i.e., published and unpublished studies and supplemental data not regularly indexed in bibliographic database sources). For example, for SRs of prescription drugs, there are multiple gray literature sources, including US Food and Drug Administration (FDA) documents, registries with results (e.g., ClinicalTrials.gov), and soliciting information from experts, the public, and pharmaceutical manufacturers. Prior evaluation of FDA gray literature sources found that more than half of registration studies submitted for FDA review remain unpublished post-drug approval [3] and that publication status was related to study results [4]. Searching and including information from FDA documents in SRs performed for the Drug Effectiveness Review Project (DERP) helped confirm previous findings, identify important harms, and fill gaps in the evidence base, as well as changed conclusion statements [5]. There is more uncertainty about the value of soliciting information from pharmaceutical manufacturers. To help address or prevent publication bias as well as to allow a transparent mechanism for manufacturer participation, both DERP and the AHRQ EPC program have instituted processes for regularly soliciting information from pharmaceutical manufacturers [1, 6]. However, concerns have been raised about the potential for increased bias in unpublished study reports from manufacturers due to the lack of peer review, as well as the associated workload. Manufacturer submissions can be quite voluminous and the manpower required to organize and manage it, such as converting submissions into a format consistent with existing processes can be significant. The potential trade-offs introduced by use of manufacturer submitted evidence, both published and unpublished, have not been evaluated.

DERP is a collaboration of state Medicaid agencies that has been commissioning SRs to inform local decision-making on prescription drugs since 2003 [7, 8]. To supplement standard comprehensive literature search methods [6], we have been soliciting both published and unpublished evidence from pharmaceutical manufacturers of brand name drugs from DERP’s inception. We refer to submitted document packets as ‘dossiers.’ Given that we have screened data submitted in dossiers for over 40 DERP SRs in that time, we have a large amount of data on the type and value of information obtained through this process.

Our purpose was to retrospectively evaluate the information obtained in these dossiers in terms of the amount and type of information, the quality of studies submitted, how evidence obtained only through these requests was used, and whether it influenced SR conclusions. To our knowledge, this is the first evaluation of dossiers. Our results may be highly relevant to the standard of practice for systematic SRs, shedding light on the value, workload, and potential pitfalls of seeking and including such evidence.

## Methods

Data submitted by pharmaceutical manufacturers was collected from “dossiers” submitted following formal requests from July 2006 to June 2015. DERP is operated in 3-year cycles, beginning in 2003 (DERP I). The dossier process was not consistently used in DERP I (2003–2006), and those data were not included. Over the period of this study (DERP II–DERP IV), there were changes in the number and type of participants, the scope of SRs, and the requirements for dossiers submitted. Briefly, DERP II focused on drug class SRs, DERP III expanded the scope to include some with multiple drug classes for specific diseases, and DERP IV implemented “streamlined” methods (e.g., including head to head evidence only) to reduce the size, cost, and timeline for SRs.

### DERP dossier request process

Brand name drugs were used to identify pharmaceutical manufacturers invited to submit a dossier to DERP. These manufacturers were sent a formal request, with protocols for the SR and for the dossier, and asked to respond within a specified timeframe (2 months in DERP II and III, 2 weeks in DERP IV). The requests were sent out immediately following finalization of the key question and inclusion/exclusion criteria for the SR.

The dossier request protocol changed over the course of DERP, starting with a fairly broad request for information and focusing down to request only head to head evidence in DERP IV. In all phases, both published and unpublished evidence, including subgroup data, was requested. We asked for a list of citations of published studies and details on study identifiers, population characteristics, outcome measures, and results for unpublished studies. Details of the dossier submission protocol are available in Additional file 1.

### Data collection

We included dossiers submitted in response to requests for 40 DERP SRs. We excluded SRs produced in the first phase of DERP (2003–2005) or SRs prepared by subcontractors because of lack of availability of complete data. We extracted the following data: review type (original or update), DERP phase, clinical area (e.g., cardiovascular), number of brand name products, number of manufacturers submitting dossiers, number of dossiers submitted, and total number of studies in dossiers per review included. For evidence included in each review we recorded the total number of studies by publication status (published or unpublished) and the number of sets of data supplemental to a published study (e.g., subgroup analyses) also by publication status. We also extracted information on study designs (e.g., head to head and placebo-controlled), whether they contributed evidence to address questions on subgroups of interest. Data were collected using a template, after multiple rounds of pilot testing, by one reviewer (ST) and spot-checked by a second for accuracy (MM and KP).

### Data analysis

We assessed evidence characteristics on a per-dossier basis including the mean number of dossiers received per review, mean number of studies submitted in dossiers (regardless of inclusion decision), proportion of all submitted studies included in SRs, study design, the proportion of unique studies versus supplemental data, the proportion of unpublished studies versus published evidence, and the quality ratings of unique studies included in a review (good, far, or poor) according to DERP methods [6]. Our quality rating tool for randomized controlled trials include assessments of randomization, allocation concealment, and blinding methods, comparability of groups at baseline, and amount and handling of missing data. These quality assessments were made at the time each SR was being conducted, by team members trained and experienced in quality assessment. All quality assessments underwent dual review, with at least one senior reviewer. We were unable to locate quality assessment records for 34 (21%) dossier studies mostly (29/34, 85%) from the early dates of this study. For these, we assigned a rating of poor if it was simply cited but not further discussed (consistent with DERP methods); otherwise, we assumed it was fair quality.

We assessed the following on a per-SR basis: the proportion of SRs including studies from a dossier and the proportion of all studies, unpublished studies, head to head studies, and studies addressing subgroups included in a SR solely identified in a dossier (versus other sources). In the DERP SRs, literature searches were conducted in consultation with a librarian using multiple electronic databases (MEDLINE, the Cochrane Library, and PsychINFO if appropriate) and searched bibliographies of included studies and FDA documents. Dual review was used. We also evaluated these outcomes by SR clinical area, original versus update SR, and by phase of DERP (II, III, and IV). For DERP IV, we evaluated dossier submissions versus searches of ClinicalTrials.gov to determine if there was publication bias. The registry was not used in DERP II and III.

We assessed the impact of the studies included from dossiers on the review conclusions. Impact was categorized into five types: (a) information was used qualitatively only, (b) information was used quantitatively (meta-analysis), (c) information provides evidence to fill a gap in the non-dossier evidence, (d) information confirms findings of non-dossier-evidence, and (e) information directly changed conclusion statements. We used these categories because as we found them to be useful in our prior work evaluating FDA documents [5]. Multiple categories could be selected as deemed appropriate, for example, a study could contribute to a meta-analysis and confirm prior findings. To further characterize the subset of studies that were found to influence conclusions, we recorded the direction of the change as better than, worse than, or no difference than the comparator drug. For evidence that was not comparative (e.g., placebo-controlled trial evidence) we used a category of “other.” For studies marked as filling a gap in other evidence, we recorded information on whether the study provided evidence on gaps in evidence specific to a population (e.g., by age or gender), outcome (e.g., providing the only evidence on an important outcome) or both. These assessments were made with dual review (see list of reviewers in the “Acknowledgements” section); differences were resolved through consensus (MM and KP).

## Results

We included 40 DERP SRs conducted between 2006 and 2015. These SRs covered 27 topics. These 40 SRs included 541 brand-name drug products made by 120 manufacturers. Solicitation of dossiers from these companies resulted in 160 dossiers being received from 41 manufacturers; a 30% response rate (Table 1). These dossiers included 7360 studies (mean 46 per dossier and 184 per review), with 160 (2.2%) of these meeting inclusion criteria for the SRs and were not identified in other searches (mean 4 per review).

**Table 1:** Characteristics of evidence submitted to DERP by manufacturers

Dossier evidence	*n*/*N* (%)
Number dossiers received	160
Number of drug products included in DERP reports	541
Number of manufacturers submitting dossiers	41
Studies/supplemental data submitted	7360
Studies/supplemental data Included from dossiers	160
Contribution of dossier evidence incremental to standard searching methods	
Proportion of submitted evidence included	160/7360 (2.2%)
Studies/supplemental data included from dossier per review (mean)	160/40 (4)
Proportion of all studies in DERP reviews from dossiers	160/2234 (7.2%)
Unique study or supplemental data	
Proportion studies included	123/160 (76.9%)
Proportion supplemental data included	37/160 (23.1%)
Unpublished studies	
Proportion of included dossier studies/supplemental data unpublished	65/160 (40.6%),
Head to head studies	
Proportion of included dossier studies	41/160 (25.6%)
Proportion of submitted studies	41/7360 (0.6%)
Proportion of all head to head studies in DERP reports from dossiers	41/536 (7.6%)
Subgroup evidence	
Proportion of studies included from dossiers with subgroup evidence	26/160 (16.3%)
Proportion of all studies in DERP reports on subgroups from dossiers	26/363 (6.7%)
Quality of studies (*n* = 123, excludes supplemental data)	
Good	9/123 (7.3%)
Fair	97/123 (78.9%)
Poor	18/123 (14.6%)

Sixty-three percent (25 of 40) of SRs included a unique study or supplemental data (or both) from a dossier submission. Considering all sources, 2234 studies were included in these 40 SRs, such that the dossier submissions contributed 7.2% of the total included studies. As an example, we conducted an update of our review on second-generation antipsychotics in 2006 that included one new brand-name drug product that was added to 17 drugs in the prior review. We received seven dossiers (one regarding the new drug), from five manufacturers. These dossiers contained 736 citations, of which 7 published studies were not identified in other searches and were included in the review.

### Characteristics of dossier evidence included in DERP SRs

Of the 160 studies or supplemental evidence added from dossiers, 77% were primary studies and 23% were supplemental data relating to previously included studies (e.g., subgroup data and analyses or additional outcomes). The majority of the studies were rated as fair quality (Table 1), with 7.3% rated good and 15% rated poor quality. One third of the 123 primary studies identified in dossiers and included in DERP SRs were head to head comparisons of included drugs. A similar proportion was placebo- or active-controlled trials, and a smaller percentage was observational studies. Out of all head to head studies included in 40 DERP SRs, 7.6% were identified only via the dossier submission process. Almost 42% of the studies identified from dossiers and included in a DERP review were unpublished, representing 2.9% of the total included studies across the SRs.

The studies identified through dossier submissions contributed information on a pre-specified subgroup of interest for the review in 16% of the cases. Out of all studies included to address subgroup issues across all 40 SRs, 6.7% came from the dossier submissions, and not through regular search methods.

### Impact of dossier evidence

Fifteen SRs, out of 40 (38%), had conclusions altered by evidence provided in a dossier (Table 2). These 15 SRs included 55 new unique studies (26 unpublished) and 9 supplemental data sets (4 unpublished) from a dossier submission. While most of the dossier evidence filled gaps in the other evidence, five SRs (involving eight unique studies) used the evidence to supplement other evidence. There were two SRs, with one unique study each, where the dossier evidence contradicted the other evidence. One was a trial of drugs for multiple sclerosis, where findings at 1.5 years in the dossier study favored the manufacturer’s drug, while other studies with 5 years of follow-up found no significant difference between drugs. The other was in the SR of long-acting insulins, where two small observational studies come to different conclusions on fetal risks with maternal use of the insulin during pregnancy. The study submitted in the dossier found slightly increased risks on a few outcomes with the competitor insulin, while the other study found no significant differences. The studies could not be pooled due to differences in reporting of outcomes.

**Table 2:** DERP SRs with changes in findings based on dossier evidence

DERP SR	*N* and type of evidence*	*N* unpublished	Fills gaps? (*N*)	Contradicts other evidence?
Atypical antipsychotics update 2	1 unique study	0	No	No
Atypical antipsychotics update 3	23 unique study 3 supplemental data	20 3	Yes (19) Yes (3)	No, included in meta-analyses
Atypical antipsychotics update 4	2 unique study 1 supplemental data	1 0	Yes (2) Yes (1)	NA
ADHD update 4	1 unique study	No	Yes (1)	NA
Asthma/COPD	5 unique study 1 supplemental data	2 0	Yes (4) Yes (1)	No, included in meta-analysis
Combination products	1 unique study	0	Yes (1)	NA
Diabetes drugs	2 unique study	0	Yes (2)	NA
Hepatitis C original	2 unique study	1	Yes (2)	NA
Hepatitis C update 1	3 unique study	1	Yes (3)	NA
Long-acting insulins	4 unique study 2 supplemental data	0 0	Yes (3) Yes (2)	Yes, findings in offspring of women using insulin during pregnancy contradict one other study found.
Multiple sclerosis update 2	1 unique study	0	No	Yes, contradicted findings of studies with longer duration (favored manufacturers drug in shorter duration, no difference with longer duration)
Neuropathic pain	1 unique study	0	Yes (1)	NA
Proton pump inhibitors Update 5	6 unique study	0	Yes (6)	NA
Topical calcineurin inhibitors	3 unique study 1 supplemental data	1 0	Yes (2) Yes (1)	NA
Triptans update 4	1 supplemental data	1	Yes (1)	NA

*Unique = new unique study, *Supplemental* = data that supplements a previously identified unique study, *NA*, not applicable

Table 3 shows how we used evidence from dossier submissions whether they had impact on the review conclusions according to the characteristics of the evidence, and the majority of studies included from dossiers were used qualitatively in narrative syntheses (poor quality studies were generally not synthesized with better evidence), with only 16% used in meta-analyses. Supplemental data were not included in any meta-analyses, while 22% of head to head trials from dossiers were used in meta-analyses. Slightly more published studies (19%) contributed to meta-analyses than did unpublished studies (12%).

**Table 3:** Dossier evidence use according to evidence characteristics

	Total included studies	Used qualitatively	Used in MA	Fills a gap	Confirms findings	Changes conclusion
All dossier evidence	160	142 (89%)	26 (16%)	72 (45%)	66 (41%)	67 (42%)
Unique studies	123	105 (85%)	24 (20%)	57 (46%)	47 (38%)	56 (46%)
Supplemental evidence	37	36 (97%)	2 (5%)	15 (41%)	19 (51%)	9 (24%)
By study design						
Head to head trial	41	32 (78%)	9 (22%)	18 (44%)	15 (37%)	21 (13%)
Placebo-controlled trial	46	39 (85%)	10 (22%)	23 (50%)	21 (46%)	21 (46%)
Observational study	28	27 (96%)	0	13 (46%)	5 (18%)	9 (32%)
Publication status						
Published (all)	95	81 (85%)	18 (19%)	43 (45%)	43 (45%)	37 (39%)
Unpublished (all)	65	58 (89%)	8 (12%)	29 (45%)	23 (35%)	29 (45%)
Unpublished unique studies	46	39 (85%)	8 (17%)	22 (48%)	14 (30%)	25 (54%)
Unpublished supplemental	19	19 (100%)	0	7 (37%)	9 (47%)	4 (21%)
Subgroup evidence	26	25 (96%)	3 (12%)	15 (58%)	9 (35%)	12 (46%)
DERP phase						
DERP II	68	62 (91%)	13 (19%)	22 (32%)	32 (47%)	16 (24%)
DERP III	50	44 (88%)	7 (14%)	28 (56%)	19 (38%)	29 (58%)
DERP IV	42	35 (83%)	6 (14%)	22 (52%)	15 (36%)	21 (50%)
Publication status by DERP phase						
Published	95					
DERP II	55	50 (91%)	9 (16%)	21 (38%)	24 (44%)	15 (27%)
DERP III	17	14 (82%)	3 (18%)	8 (47%)	9 (53%)	7 (41%)
DERP IV	23	17 (74%)	6 (26%)	14 (61%)	10 (43%)	15 (65%)
Unpublished	65					
DERP II	13	9 (69%)	4 (31%)	1 (8%)	8 (62%)	1 (8%)
DERP III	33	30 (91%)	4 (12%)	20 (61%)	10 (30%)	22 (67%)
DERP IV	19	19 (100%)	0	8 (42%)	5 (26%)	6 (32%)
Report type	160					
Original	48	39 (81%)	15 (31%)	19 (40%)	29 (60%)	23 (48%)
Update	112	100 (89%)	11 (10%)	53 (47%)	37 (33%)	43 (38%)

The dossier studies were used to fill a gap in the evidence 45% of the time, with 58% of the gaps related to subpopulations. No dossier evidence was used to fill a gap in outcomes. Analysis of study design or publication status did not alter these findings importantly. Fifty-one percent of the supplemental evidence submitted and included confirmed the findings of other published studies, with a higher proportion of placebo-controlled trials confirming findings (46%) than head to head (37%) or observational studies (18%). Unpublished studies confirmed findings of other published studies less often, 30% of the time.

The dossier studies resulted in changes to conclusion statements in 42% of the SRs, with new primary studies altering conclusions more frequently than supplemental evidence submissions. More of these impacts on conclusion statements came from placebo-controlled trials than from head to head trials, and few from observational studies. Placebo-controlled trial evidence added conclusions, rather than changing conclusions. For example, there was often inadequate head to head evidence in children, and in one case the only conclusions on the antipsychotic drug quetiapine in children with bipolar disorder were comparisons with placebo. Unpublished evidence changed conclusions in 29 of 65 instances (45%), and supplemental evidence altered conclusions 24% of the time (9 of 37 instances).

Dossier evidence that was used to address questions in pre-specified subgroups was used to fill a gap in the evidence 58% of the time, with all of these being gaps in population subgroups. This evidence affected conclusions almost half of the time.

### Temporal trends

The number of dossiers received per review conducted increased across the three DERP phases, and a larger proportion of submitted studies were ultimately included in DERP III and IV (4.4% and 3.6%) compared with DERP II (1.3%) (Table 4). The proportion of DERP SRs with dossier evidence included (versus no dossier evidence) also increased across the DERP phases. Considering the proportion of studies rated good and poor, the quality of studies included from dossiers improved across DERP phases.

**Table 4:** Temporal trends in dossier evidence inclusion and characteristics

	DERP II	DERP III	DERP IV
Proportion of SRs with dossier data	11/21 (52%)	7/10 (70%)	7/9 (78%)
Proportion of total dossier evidence	68/160 (42.5%)	50/160 (31.3%)	42/160 (26.3%)
Studies vs supplemental evidence			
Studies	60	37	26
Supplemental evidence	8	13	16
Study quality			
Good	4 (6.7%)	1 (2.7%)	4 (15.4%)
Fair	43 (71.7%)	33 (89.2%)	20 (76.9%)
Poor	13 (21.7%)	3 (8.1%)	2 (7.7%)

Fewer studies included from dossiers influenced conclusions in DERP II (24%) than in DERP III and IV (58% and 50%). The proportion of published studies that contributed to conclusion statements increased over the DERP phases, but unpublished studies did not follow this pattern, with the greatest proportion influencing conclusions in DERP III. The inclusion and influence of studies and supplemental evidence remained constant across the phases. According to DERP phase (II, III, and IV), a higher proportion of dossier studies filled a gap in evidence and resulted in changes to conclusions in DERP III and IV than in DERP II. Updates of SRs had a slightly greater mean number of dossiers submitted (4.1% versus 3.3%) and proportion of submitted studies included (2.3% versus 2.0%) compared with original SRs.

### Clinical categories

Looking at the impact of dossier evidence by clinical category; the category with the largest number of studies included from dossiers was psychiatric topics, but this was only 6.8% of the total studies included in these SRs. The two gastroenterological topics included a larger proportion of the total included studies (22%) and on a per-SR basis (Appendix 1). The studies included in the psychiatric topics affected conclusions slightly more often than those added to the gastrointestinal topics (39% versus 32%). No studies were included from dossiers in three DERP SRs on cardiovascular drugs (antiplatelets, statins, and novel anticoagulants). Considering the quality of evidence from dossiers, the studies included in two SRs on drugs for hepatitis C had more good quality studies (38%) and SRs on psychiatric drugs had the fewest (4.3%). The proportion of poor-quality studies from dossiers ranged from 11% (psychiatric topics) to 28% (respiratory topics).

Psychiatric, endocrine, and dermatology topics had the greatest proportion of included manufacturer submitted studies affecting conclusions, with at least half of the studies submitted changing a conclusion, and psychiatric, endocrine, and gastroenterology had the greatest proportion of submitted studies filling a gap in the non-dossier evidence (Appendix 2). Supplemental evidence (*N* = 37) was not used in meta-analyses for any clinical category, but was used to fill a gap 50% of the time for psychiatric, endocrine, neurology/pain, and respiratory topics and for the single instance for a gastroenterological topic. Supplemental evidence influenced conclusions half of the time for endocrine, psychiatric, and respiratory topics. For unpublished evidence included from dossiers (*N* = 65), a third were used in meta-analyses in neurology or pain topics, and 13% in psychiatric topics. Unpublished evidence was used to fill a gap in evidence and altered conclusions in psychiatric topics 67% and 77% of the time and in hepatitis topics 40% and 20% of the time, respectively.

### Direction of influence on conclusions and reporting bias

We further evaluated the direction that the studies included from dossiers influenced the conclusions (Table 5). To do this, we considered the direction of influence of new evidence from a dossier (favoring the manufacturer’s drug, the competitor drug, or no change) in relation to the conclusion that would have been drawn without the evidence. This includes evidence in prior SRs (for updates) as well as other new evidence for the current review. We selected two groups of studies with potential for influencing conclusions in this dossier process to evaluate further: unpublished studies, because they have not gone through a peer review process for publication, and studies of psychiatric topics because we found that they had the highest frequency of changing conclusions.

**Table 5:** Direction of influence on SR conclusions

	Total affecting conclusions	Favors dossier drug	Favors a competitor	No difference	Other
Unpublished	25	13 (52%)	2 (8.0%)	10 (40%)	2 (8.0%)
Psychiatry topics	31	15 (48%)	6 (19%)	13 (42%)	2 (6%)

Over half of the unpublished studies included from dossiers influenced the conclusions in favor of the manufacturer’s drug for at least one outcome and patient population, compared with 8.0% influencing the conclusion in a direction that favored a competitor drug and 40% finding no significant benefit, depending on the outcome and/or population. Further examination of these examples finds that 92% of dossier studies affecting conclusions in favor of the submitting manufacturer’s drug were the only evidence for the drug in a specific population or outcome, and 85% were placebo-controlled trials.

For the psychiatric topic SRs, close to half the studies included from dossiers resulted in conclusions that favored the manufacturer’s drug, but in this case 19% resulted in favoring a competing drug. In this set of studies, we also found that most (86%) of those affecting a conclusion in favor of the submitting manufacturer’s drug were the only evidence for that drug in a population or for a key outcome (e.g., weight gain), and that 93% were placebo-controlled trials.

Finally, we evaluated the difference between the manufacturer submitted studies and the ClinicalTrials.gov registry for DERP IV SRs as an indicator of reporting bias (i.e., lack of reporting entire studies). We found no evidence of studies in ClinicaTrials.gov that were not submitted through the dossier process, with the exception of a few studies on Hepatitis C drugs that did not fit the usual pattern for DERP inclusion criteria. For this review, head to head comparisons of drug regimens were included, rather than limiting to head to head comparisons of specific drugs as is typical for DERP SRs. While eligibility of these study designs was described in the inclusion criteria, the protocol for dossier submissions refers to head to head comparisons only and as such, it may have been less clear to the manufacturers that these studies were eligible for submission.

## Discussion

This is the first evaluation of a formal process to request evidence from pharmaceutical companies on the benefits and harms of their drugs in the context of conducting SRs intended to directly inform decision-making. The purpose of seeking out evidence from drug manufacturers to supplement literature searches (i.e., dossiers) is to reduce reporting and publication bias and encourage transparency and participation from industry. However, concerns have been raised about the usability of the information provided and the associated workload. Our evaluation of this practice, from 9 years of DERP experience, provides greatly needed information about the trade-offs of this process.

Manufacturer dossiers contributed important information to a majority of DERP SRs (63%) and were associated with a manageable workload (i.e., mean of 184 additional studies screened per review). Although only a small proportion of the materials submitted were ultimately incorporated in our SRs (2.2%), they contributed to filling gaps in evidence and in forming new conclusions where none could be made without this evidence. Dossiers identified published studies missed by our standard search process, and provided earlier access to influential data that was likely soon to be published. Typically, unpublished studies were included because they were the first to examine the use of a drug in a given population (e.g. quetiapine for patients with depression) or to report a given outcome and therefore resulted in new conclusion statements.

Although concerns have been raised about the potential for increased bias in unpublished study reports due to the lack of peer review [9], we found the quality of the unpublished study reports submitted in dossiers to be mostly similar to that of the overall quality in DERP SRs, although a smaller proportion were rated poor quality (i.e., 22% vs. 7.7%). Unpublished studies would typically be expected to have “negative” findings; those submitted through DERP dossiers actually favored the manufacturer’s drug more than half of the time. A simple reason for this could be that the unpublished studies submitted to DERP were relatively better than expected because they were soon to be published. A more concerning possibility is that the submitted studies were a biased selection of the total group of unpublished studies.

We found that the relevancy of dossier material submitted as shown by the proportion of DERP SRs including evidence from dossiers increased over time (52% in DERP II, 70% in DERP III, and 78% in DERP IV). The increase in the number of studies included from dossiers in DERP SRs over time could reflect increased desire of the pharmaceutical manufacturers to have evidence on their drugs included in a DERP SR, improved outreach, and/or improved clarity of the submission protocol over time.

Our findings are consistent with previous related work that found similar value in using FDA documents to supplement literature searches [5, 10]. Other related work includes evaluations of the quality of economic evaluations submitted to NICE (the National Institute for Clinical Excellence) [11], reporting bias in studies of drugs submitted to the FDA [12], public trial registries [13], and various methodologies intended to reduce publication bias [14], or to identify unpublished evidence for SRs [15–17]. These studies find that reporting bias is a serious issue but not simple to predict or to determine the extent of the problem across broad intervention and population groups. A recent study evaluated the impact of searching for additional evidence in trial registries on SRs [18]. While new data had some influence on pooled point estimates, the authors conclude that the new studies did not change the interpretation of the results.

Limitations of our study are mainly related to the type of data we collected and the applicability of our findings to other SR groups. First, although our dataset was designed for simple aquisition and validation of data, the accuracy of some data is uncertain. An example is the total number of citations submitted in dossiers in the earlier years when we frequently received very large volumes of evidence—some provided only in paper versions may not have been counted, and duplicate submissions may have been double counted. Second, as we did not include dossier data on SRs produced by a subcontracting EPC (10 SRs) because we had not asked them to collect these data, it is possible they had different experiences and including their data may change our conclusions. Third, although we reported on the average *volume* of studies, which can provide some general information about workload, we did not formally collect data on additional workload demands unique to processing dossier materials including organization and management of the data submitted, and converting submissions into a format consistent with our own processes for comparison. Fourth, we also do not have information on why we missed published studies in our standard search processes, and what the characteristics of these missed studies were.

The applicability of our results to other SRs will depend on the similarity of scope and purpose, with our results being most applicable to SRs of drug interventions; however, our experiences may be generalizable in terms of the key concepts. Our work raises new questions that could be addressed in future research. Key among these is the need for further analyses of manufacturer submissions in the context of other sources, such as trial registries. There is also need for further examination of the quality of the studies included from submissions, particularly unpublished studies to further examine potential bias of submitted evidence.

## Conclusions

In SRs conducted for the Drug Effectiveness Review Project (DERP), requesting evidence from pharmaceutical manufacturers helped to reduce reporting and publication bias and helped to fill important gaps, sometimes leading to new or altered conclusions, primarily where no other evidence existed. The use of dossiers in DERP SRs potentially improved transparency and participation from industry stakeholders.

### Additional file

Additional file 1: The Drug Effectiveness review project evidence submission protocol. (PDF 180 kb)

## Appendix 1

**Table 6:** Dossier unique study evidence by disease category (total = 123)

Clinical category	Number of reports	Total studies (mean/review)	Study quality		
			Good	Fair	Poor
Cardiovascular	3	0 (0)	–	–	–
Dermatology	1	6 (6)	–	6 (100%)	–
Endocrinology	3	12 (4)	–	10 (10.4%)	2 (17%)
Gastrointestinal	2	22 (11)	1	18 (19%)	3
Hepatitis	2	8 (4)	3 (38%)	4 (50%)	1 (13%)
Neurology/pain	5	11 (2)	2 (18%)	7 (64%)	2 (18%)
Psychiatry	6	46 (8)	2 (4.3%)	39 (85%)	5 (11%)
Respiratory	3	18 (9)	1 (5.6%)	12 (67%)	5 (27.8%)

## Appendix 2

**Table 7:** Impact of dossier evidence on DERP reports by clinical category

Clinical area	Included from dossiers	Used qualitatively	Used in MA	Gap	Confirms	Changes
All	160	142(89%)	26 (16%)	72 (45%)	66 (41%)	67 (42%)
Cardiovascular	3	3(100%)	0	0	3(100%)	0
Dermatology	10	10 (100%)	6(60%)	3(30%)	10 (100%)	5(50%)
Endocrinology	16	11(69%)	4(25%)	8(50%)	9(56%)	9(56%)
Gastrointestinal	22	21(95%)	1(5%)	12(55%)	6(27%)	6(27%)
Hepatitis	17	15 (88%)	2(12%)	8(47%)	7(41%)	5(29%)
Pain/neurology	17	12(71%)	5(29%)	4(24%)	11(65%)	4(24%)
Psychiatry	54	49 (91%)	6(11%)	29(54%)	15(28%)	31(57%)
Respiratory	20	19(95%)	1(5%)	6(30%)	5(25%)	6(30%)
Studies	123					
Cardiovascular	0	–	–	–	–	
Dermatology	6	6 (100%)	5 (83%)	2 (33%)	6 (100%)	4 (67%)
Endocrinology	12	8 (67%)	4 (33%)	6 (50%)	7 (58%)	7 (58%)
Gastrointestinal	21	20 (95%)	1 (5%)	11 (52%)	6 (29%)	6 (6%)
Hepatitis	8	6 (75%)	2 (25%)	5 (63%)	2 (25%)	5 (63%)
Neurology/pain	11	6 (55%)	5 (45%)	1 (9%)	7 (64%)	2 (18%)
Psychiatry	46	41 (89%)	6 (13%)	26 (57%)	14 (30%)	27 (59%)
Respiratory	19	18 (95%)	1(5%)	6 (32%)	5 (26%)	5 (26%)
Supplemental evidence	37					
Cardiovascular	3	3 (100%)	0	0	3(100%)	0
Dermatology	4	4(100%)	2(50%)	1 (25%)	4(100%)	1 (25%)
Endocrinology	4	4(100%)	0	2 (50%)	2(50%)	2 (50%)
Gastrointestinal	1	1 (100%)	0	1(100%)	0	0
Hepatitis	9	9 (100%)	0	3 (33%)	5 (56%)	0
Neurology/pain	6	6 (100%)	0	3(50%)	4 (67%)	1 (17%)
Psychiatry	8	8 (100%)	0	4 (50%)	1 (13%)	4 (50%)
Respiratory	2	2 (100%)	0	1 (50%)	0	1(50%)
Published studies	95					
Cardiovascular	3	3 (100%)	0	0	3 (100%)	0
Dermatology	10	10 (100%)	7 (70%)	3 (30%)	10 (100%)	5 (50%)
Endocrinology	15	10 (67%)	4 (27%)	8 (53%)	8 (53%)	9 (60%)
Gastrointestinal	22	20 (91%)	1 (5%)	12 (55%)	6 (27%)	6 (27%)
Hepatitis	7	5 (71%)	2 (29%)	4 (57%)	3(43%)	3 (43%)
Neurology	5	4 (80%)	1 (20%)	2 (40%)	2 (40%)	3 (30%)
Psychiatry	24	21 (88%)	2 (8%)	10 (42%)	8 (33%)	8 (33%)
Respiratory	9	8 (89%)	1 (11%)	4 (44%)	3 (33%)	3 (33%)
Unpublished evidence	65					
Cardiovascular	0	–	–	–	–	
Dermatology	0	–	–	–	–	–
Endocrinology	1	1 (100%)	0	0	1(100%)	0
Gastrointestinal	0	–	–	–	–	–
Hepatitis	10	10 (100%)	0	4 (40%)	4 (40%)	2 (20%)
Neurology	12	8 (67%)	4 (33%)	2 (17%)	9 (75%)	1 (8%)
Psychiatry	30	27 (90%)	4 (13%)	20 (67%)	7 (23%)	23 (77%)
Respiratory	12	12 (100%)	0	3 (25%)	2 (17%)	3 (25%)

*MA* meta-analyses

## Abbreviations

AHRQ: Agency for Healthcare Research and Quality
DERP: Drug Effectiveness Review Project
EPC: Evidence-based Practice Center
FDA: US Food and Drug Administration
