# Supplementary material for: A study of the value of requesting information from drug manufacturers for systematic reviews; 9 years of experience from the drug effectiveness review project
Source: Syst Rev. 2018 Oct 22;7:172. doi: 10.1186/s13643-018-0834-2 (PMC6198528; doi:10.1186/s13643-018-0834-2)
Supplement: Supplementary file 1 — The Drug Effectiveness review project evidence submission protocol. (PDF 180 kb) [file 13643_2018_834_MOESM1_ESM.pdf]

THE DRUG EFFECTIVENESS REVIEW PROJECT
EVIDENCE SUBMISSION PROTOCOL

A FORMAT FOR SUBMISSION OF CLINICAL
EVIDENCE FOR SYSTEMATIC
EVIDENCE-BASED REVIEWS OF DRUG CLASSES

The Center for Evidence-based Policy
Oregon Health & Science University

Version 2.5

July 2010

Thanks to participants in the Project and pharmaceutical companies for their suggestions.

ALL INFORMATION SUBMITTED TO THE CENTER FOR EVIDENCE-BASED POLICY MAY BE AVAILABLE TO THE PUBLIC. ANY AND ALL MARKINGS OR STATEMENTS OF CONFIDENTIALITY SHALL BE CONSIDERED NULL AND VOID.

TABLE OF CONTENTS
EVIDENCE SUBMISSION PROTOCOL

I. Overview of the Drug Effectiveness Review Project 3

II. About the Evidence Submission Protocol ……….. 3

A. Information for Responders 3

B. How to Submit ………………………….. 4

III. Details of the Evidence Submission 5

A. Product Information ………………………………………………......... 5

B. Supporting Clinical Studies ………………................................. 5

C. Supplemental Data for Published Clinical Studies …………........ 7

Appendix: Evidence Submission Form ……………………………………….. 8

I. THE DRUG EFFECTIVENESS REVIEW PROJECT

The Drug Effectiveness Review Project (DERP) is a collaboration of public organizations that have joined together to provide systematic evidence-based reviews of the comparative effectiveness and harms of drugs in many widely used drug classes, and to apply those findings to inform public policy. Participants are committed to using evidence-based principles for drug coverage decisions. They believe that drug products should be subjected to a rigorous clinical review based on evidence from the clinical literature. Evaluating the efficacy, harms, and effectiveness of drugs is the foundation of the Project. Where feasible, evidence evaluating how drug products directly compare to one another are preferred over comparisons to placebo.

The goal of the Drug Effectiveness Review Project is optimal patient care, taking into account the reality of constrained resources. It is intended to shift emphasis away from the drug price/rebate approach typically utilized for drug coverage decisions to an evidence-based approach to health care delivery. Simply stated, the intent is for manufacturers to provide evidence of the value of their products compared to similar products in terms of health outcomes for consumers. Establishing the comparative effectiveness of drugs will allow participating organizations to make informed purchasing decisions with value in mind.

The Drug Effectiveness Review Project is organized by the Center for Evidence-based Policy, Oregon Health & Science University. The Center collaborates with the Evidence-based Practice Center (EPC) at Oregon Health & Science University, who coordinates production of all systematic reviews.

II. ABOUT THE DRUG EFFECTIVENESS REVIEW PROJECT SUBMISSION PROTOCOL

The purpose of this request for information is to identify all the scientific information pertinent to the key questions, including both published and unpublished data. The goal is to identify where evidence regarding effectiveness and harms exists and where it is absent. This submission protocol supports the review process by standardizing information requirements and communicating key elements of the review, including key questions. This protocol is unique to the Drug Effectiveness Review Project. It is not the same as that currently used by the State of Oregon or by the Academy of Managed Care Pharmacy (AMCP). Evidence submissions are logged by the Center for Evidence Based Policy and distributed to the EPC responsible for conducting the systematic review.

<u>Please Note:</u> The cut off for new drugs to be included in a DERP review is the same date that dossier submissions are due. After this date has passed new drugs will be acknowledged in the report, but not included in the drug class review.

A. INFORMATION FOR RESPONDERS

Manufacturers should understand that submission of information in the format recommended herein does not mean a product will be listed as a preferred agent in any purchasing or information process. This document describes the minimum information requirements necessary to support a comprehensive assessment of the product in relation to other similar products.

This is a voluntary and unsolicited request for information. All costs for complying with this request must be born by the submitter. This protocol has been determined to be sufficient for the purposes of the Drug Effectiveness Review Project evaluation. The Project cannot guarantee that a submission that is incomplete or fails to follow this protocol will be considered. Likewise, provision of excess information outside that outlined in the protocol in Section III may jeopardize the ability of reviewers to consider the relevant information in the submission.

No information related to price, cost, cost effectiveness or rebates is requested. Any information related to price, cost, cost effectiveness or rebates will not be reviewed.

All information submitted to the Center for Evidence-based Policy under this protocol will become available to the public. **Upon request, all information submitted to the Center will be available to the public at cost upon the release of the related systematic review or updated review.** It is the submitter's responsibility to limit information (including but not limited to clinical, price, financial and all other data and information) submitted under this protocol to data and information that may be publicly disclosed. While financial information is not requested, if it is submitted it will be available to the public.

By sending and submitting a dossier to the Center for Evidence-based Policy, regardless of any markings or statements of confidentiality contained in or on the dossier, the Company authorizes the Center to:

- **use any or all of the information in the dossier in reports (public or not),**
- **make any and all information in the dossier available to the public, and**
- **send a copy of the entire dossier to any individual or entity requesting the dossier from the Center.**

The Center shall accept all dossiers and shall have no obligation to return submitted dossiers to the company, irrespective of any markings or statements of confidentiality contained in or on the dossier.

Requests for copies of submissions can be submitted to Kathryn Clark at clarkath@ohsu.edu. Paper copies of studies from journals with copyright restrictions will not be provided to the public.

Key questions regarding the drugs to be reviewed are included with this solicitation. Information that is not relevant to these questions will not be considered.

B. HOW TO SUBMIT

The manufacturers of products identified for these reviews are required to submit clinical evidence electronically. Electronic copies of the submission should be e-mailed to Dr. Little. Please note that presently the Center is unable to accept zip files. Please e-mail all files as separate attachments when submitting.

The Center for Evidence-based Policy
Oregon Health & Science University
Alison Little MD, MPH
Medical Director, Drug Effectiveness Review Project
littleal@ohsu.edu
3455 SW US Veterans Hospital Road, Mailstop SN-4N
Portland, Oregon 97239-2941
Phone: (503) 494-7239
Fax: (503) 494-3807

Evidence submissions prepared under this protocol are submitted to and a copy retained by the Center. **Information included in any submission is subject to use and/or reference in the report. As such, this information will be shared among participating organizations and the public.** Submitters are requested not to contact the EPC, any contracting EPC, or any participating organization about the particulars or contents of submissions – all such contacts should be directed to the Medical Director for DERP, Center for Evidence-based Policy. The Center, EPC and participating organizations cannot guarantee that information submitted outside this process will be included.

As a reminder, electronic submissions are required.

III. DETAILS OF THE DRUG EFFECTIVENESS REVIEW EVIDENCE SUBMISSION

The Evidence Submission Form attached to this protocol must be used to submit information to the Center. Additional information, other than the Drug Product Label, is not requested and will not be reviewed. Specifically, please refrain from submitting AMCP dossiers, drug monographs, or other such pre-made materials which generally include a broader base of information on the epidemiology, pathophysiology, diagnosis, clinical course, and burden of the disease. In

addition, please provide a cover letter with signature verifying the accuracy and veracity of the document, and a contact person who can answer questions and provide additional information regarding the submission materials. **All AMCP dossiers will not be considered and the information submitted will not be included in the review**.

A. PRODUCT INFORMATION
Please provide the most recent FDA-approved Drug Product Label.

B. SUPPORTING CLINICAL STUDIES
Using the attached form, please submit a list of citations for all relevant published and unpublished clinical studies evaluating the harms and clinical effectiveness or efficacy of the drug(s) listed in the inclusion criteria (see attached Key Question document) that is(are) licensed by your company. When considering studies for submission, please also take into account the relevancy of the population(s) and outcome(s) to the associated criteria outlined in the Key Questions. You may provide studies of other included drugs (not licensed by your company) if you wish, although we ask that these be clearly separated from studies of your company's drug(s). Study results available only as abstracts are generally not included in DERP reports, but may be considered only if adequate additional information is submitted as unpublished study data (see below). Additionally, pharmacokinetic, pharmacodynamic and dose-ranging studies (without a placebo control) are not included – please refer to the inclusion criteria associated with the Key Questions to see what study designs and outcomes are included in this review. Specifically, please provide the following:

1. Published studies: Please provide a list of citations for all published studies that meet our review inclusion criteria for population, outcome, comparison and design.

2. Unpublished studies: We are also interested in unpublished studies (trials or observational designs) for which you may have data. For these, we ask that you submit the following information:
   - Study identifier (ClinicalTrials.gov identifier; other trial registry identification number/ name; and/or protocol number)
   - Study dates, location(s)
   - Design
   - Indication (disease state(s))
   - Patient population description
     - Mean age
     - Proportions by race
     - Proportions by sex
     - Baseline characteristics, particularly those relevant to outcomes
   - Inclusion / exclusion criteria
   - Treatment of interest and all comparisons dose, regimen, duration of treatment and follow-up

- N per arm (screened, eligible, enrolled)
- Outcomes measured
- Results
  - N per group analyzed, N lost to follow up, withdrawn overall and due to adverse events
  - Results by outcome, including adverse events
  - Statistical analysis of results – particularly comparative analyses
  - Subgroup analyses

In order to include data from unpublished studies, however, you also must submit a sufficient amount of detail on their methods to allow for adequate assessment of study quality using the criteria listed below. Data that does not meet these requirements will not be included in the report. Data submitted will become public in that others may request a copy of the information submitted, including these data.

Quality assessment criteria for controlled trials:

- Was the assignment to the treatment groups really random?
- Was the treatment allocation concealed?
- Were the groups similar at baseline in terms of prognostic factors?
- Were the eligibility criteria specified?
- Were outcome assessors blinded to the treatment allocation?
- Was the care provider blinded?
- Was the patient kept unaware of the treatment received?
- Was an intention-to-treat (ITT) analysis conducted, or was data provided from which ITT results could be calculated (i.e., number assigned to each group, number of subjects who finished in each group, and their results)?
- Did the study maintain comparable groups? Were there post-randomization *exclusions* of patients with specific characteristics?
- Was attrition, crossovers, adherence, and/or contamination reported?
- Was there differential loss to followup or overall high loss to followup?

Quality assessment criteria for non-randomized studies (observational studies)

- Was the selection of patients for inclusion non-biased (Was any group of patients systematically excluded)? For cohort studies, was an inception cohort identified?
- Is there important differential loss to followup or overall high loss to followup?
- Were the outcomes investigated specified and defined?
- Was there a clear description of the techniques used to identify the outcomes?

- Was there non-biased and accurate ascertainment of outcomes (independent ascertainers; validation of ascertainment technique)?
- Were potential confounding variables and risk factors identified and examined using acceptable statistical techniques? Did the duration of followup correlate to reasonable timing for investigated events? (Does it meet the stated threshold?)

To better understand how the answers to the above questions affect the quality assessment of a study, please refer to the Quality Assessment Methods document available on our website at http://www.ohsu.edu/ohsuedu/research/policycenter/DERP/about/methods.cfm

C. Unpublished, supplemental data for published clinical studies: We are also interested in unpublished, supplemental data pertaining to published clinical studies. Examples of this include additional detail about study methods, additional outcomes, and results of additional subgroup analyses that, for one reason or another, did not appear in the publication. Please note that we will not include results from supplemental analyses that do not provide the basis for valid, reliable and meaningful interpretations.

DRUG EFFECTIVENESS REVIEW PROJECT EVIDENCE SUBMISSION FORM

**Contact Information**	
**Manufacturer:**	ABC Inc.
**Name/Title of individual submitting information:**	Jane Doe, PharmD/Drug Information Specialist
**Email address:**	**doe@ABCcompany.com**
**Phone:**	**(123) 456-7890**

A. **Published clinical studies:** Please list below the citations for all published clinical studies of your product that are eligible for inclusion based on criteria specified in the Key Question document regarding population, outcome and study design.

B. **Unpublished clinical studies**: Below please provide a detailed description of methods and results of any unpublished clinical studies of your product(s) that are eligible for inclusion based on criteria specified in the Key Question document regarding population, outcome and study design. Please see Evidence Submission Protocol for requested data elements, and please be sure to include which registry the study is registered in, and the trial registration number.

C. **Unpublished, supplemental data for published clinical studies** (e.g., additional detail about study methods or additional outcomes that did not appear in the publication. Please only include additional information if it addresses the key questions of the report):

Example #1: additional detail about study methods

**Publication:**	Doe, JJ. Randomized, controlled trial of Drug A compared to Drug B in adults with hypertension. *ABC Journal.* 1987;5(1): 1-10.
**Supplemental methodology details:**<br>Randomization method: computer-generated randomization list *(Relevant when publication only stated, "Patients were randomized to Drug A or Drug B…")*	

Example of supplemental outcome data:

**Publication:**	Doe, JJ. Randomized, controlled trial of Drug A compared to Drug B in adults with hypertension. *ABC Journal.* 1987;5(1): 1-10.
**Supplemental outcome data (e.g., unpublished subgroup analyses, outcomes):**	

Subgroup analyses of Response Rates:			
Subgroup	Drug A<br>n/N (%)	Drug B<br>n/N (%)	P-value for comparison of Drug A and Drug B (Fisher's exact test)
Type II Diabetes			
Yes	375/500 (75%)	150/500 (30%)	<0.0001
No	350/500 (70%)	325/500 (65%)	0.0921
